# Phylogeography of the Sponge *Suberites diversicolor* in Indonesia: Insights into the Evolution of Marine Lake Populations

**DOI:** 10.1371/journal.pone.0075996

**Published:** 2013-10-01

**Authors:** Leontine E. Becking, Dirk Erpenbeck, Katja T. C. A. Peijnenburg, Nicole J. de Voogd

**Affiliations:** 1 Naturalis Biodiversity Center, Department Marine Zoology, Leiden, The Netherlands; 2 Institute for Marine Resources and Ecosystem Studies (IMARES), Maritime Department, Den Helder, The Netherlands; 3 Department of Earth- and Environmental Sciences, Palaeontology & Geobiology & GeoBio-Center, Ludwig-Maximilians-University, Munich, Germany; 4 Institute for Biodiversity and Ecosystem Dynamics (IBED), University of Amsterdam, Amsterdam, The Netherlands; University of Genova, Italy, Italy

## Abstract

The existence of multiple independently derived populations in landlocked marine lakes provides an opportunity for fundamental research into the role of isolation in population divergence and speciation in marine taxa. Marine lakes are landlocked water bodies that maintain a marine character through narrow submarine connections to the sea and could be regarded as the marine equivalents of terrestrial islands. The sponge *Suberites diversicolor* (Porifera: Demospongiae: Suberitidae) is typical of marine lake habitats in the Indo-Australian Archipelago. Four molecular markers (two mitochondrial and two nuclear) were employed to study genetic structure of populations within and between marine lakes in Indonesia and three coastal locations in Indonesia, Singapore and Australia. Within populations of *S. diversicolor* two strongly divergent lineages (A & B) (COI: *p* = 0.4% and ITS: *p* = 7.3%) were found, that may constitute cryptic species. Lineage A only occurred in Kakaban lake (East Kalimantan), while lineage B was present in all sampled populations. Within lineage B, we found low levels of genetic diversity in lakes, though there was spatial genetic population structuring. The Australian population is genetically differentiated from the Indonesian populations. Within Indonesia we did not record an East-West barrier, which has frequently been reported for other marine invertebrates. Kakaban lake is the largest and most isolated marine lake in Indonesia and contains the highest genetic diversity with genetic variants not observed elsewhere. Kakaban lake may be an area where multiple putative refugia populations have come into secondary contact, resulting in high levels of genetic diversity and a high number of endemic species.

## Introduction

**Figure 1:**
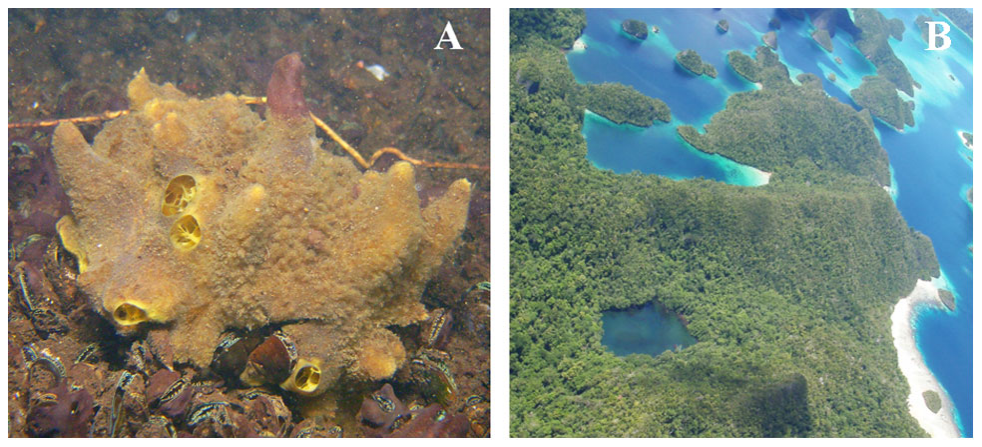
A *Suberites diversicolor* purple color morph. B Landlocked marine lakes in Raja Ampat Indonesia.

It has long been hypothesized that marine species have large geographic ranges with large population sizes, and are faced with weaker barriers to dispersal than terrestrial organisms, thus resulting in relatively slow rates of speciation (e.g. [1]). The assumed presence of circum-tropical species has supported this view. However, recent phylogeographic and population genetic studies on marine taxa portray a situation of ecologically heterogeneous environments on small spatial scales with several morphologically cryptic species instead of cosmopolitan species (e.g. [2], [3], [4], [5], [6], [7], [8]). These results suggest that there may be many more barriers to dispersal at small spatial scales than we are able to observe [1], [9], [10]. The existence of multiple independently derived populations in landlocked marine lakes provides an opportunity for fundamental research into the role of isolation in population divergence and speciation in marine taxa [11]. Marine lakes are anchialine systems, which are landlocked water bodies that maintain a marine character through narrow submarine connections to the sea (Fig. 1; [12]). The marine lakes share many characteristics with island systems [13]: they are well-defined geographically [14], [15], [16], harbor unique biota with high endemism and/or an abundance of species rare that are elsewhere [16], [17], [18], [19], [20], [21], and isolated populations [11], [22], [23]. The marine lakes in the Indo-Pacific were formed less than 12,000 years ago [13], [24], yet their biodiversity is unique. Consistent with island biogeography theory [25], [26], [27] larger lakes harbor more species than smaller ones and the most isolated lakes contain the highest number of putative endemics, while the more connected lakes are dominated by reef species [15], [16], [19]. The degree of isolation thus appears to influence the species diversity within the lakes. In the present study our overall aim was to obtain insight into the role of isolation on the genetic diversity of marine lake populations.

Phylogeographic studies of anchialine systems across the world typically show high levels of genetic differentiation between marine lake populations, suggesting little to no gene flow at small spatial scales ranging from 10 to 100 km (Table 1). Furthermore, molecular markers revealed the presence of highly divergent, but morphologically cryptic species in a number of taxa such as cnidarians, crustaceans, fish and mollusks [11], [22], [23], [28], [29], [30], [31], [32]. There are, however, exceptions to this general pattern, which have been interpreted as resulting from life history strategies involving greater dispersal capabilities [33] or too limited sampling [34].

**Table 1:** Overview of published genetic variation in populations within anchialine systems.

Anchialine system	Location	Taxon	Marker(s)	Structure	Scale of differentiation	Reference
Lake	Palau	*Mastigias papua*	mtDNA COI & nDNA ITS	each lake private haplotypes	1–50 km	[11], [59]
Lake	Palau	*Brachidontes* sp.	mtDNA COI	divergent species; each lake private haplotypes	1–50 km	[22]
Lake	Palau	*Sphaeramia orbicularis*	mtDNA control region	lakes reduced diversity private haplotypes	1–50 km	[23]
Pool	Hawaii island	*Holocaridina rubra*	mtDNA COI	each pool private haplotypes	30–50 km	[28]
Pool	Hawaii Archipelago	*Halocaridina rubra*	mtDNA COI	each pool private haplotypes	10–50 km	[29]
Pool	Maui &Hawaii	*Halocaridina rubra*	mtDNA COI	each pool private haplotypes	1–100 km	[94]
Pool	Hawaii Archipelago	*Metabenaeus lohena*	mtDNA COI	panmixia	25–300 km	[33]
Cave	Philippines	*Neritilia cavernicola*	mtDNA COI	panmixia	200 km	[34]
Cave	Australia	*Stygiocaris lancifera*	mtDNA COI 16S	divergent species	10–100 km	[30]
Cave	Spain	*Metacrangonyx longipes*	mtDNA COI 16S histone	divergent species	20–100 km	[32]
Cave	Mexico	*Creaseria morleyi*	mtDNA COI 16S	divergent populations	10–100 km	[31]

**Figure 2:**
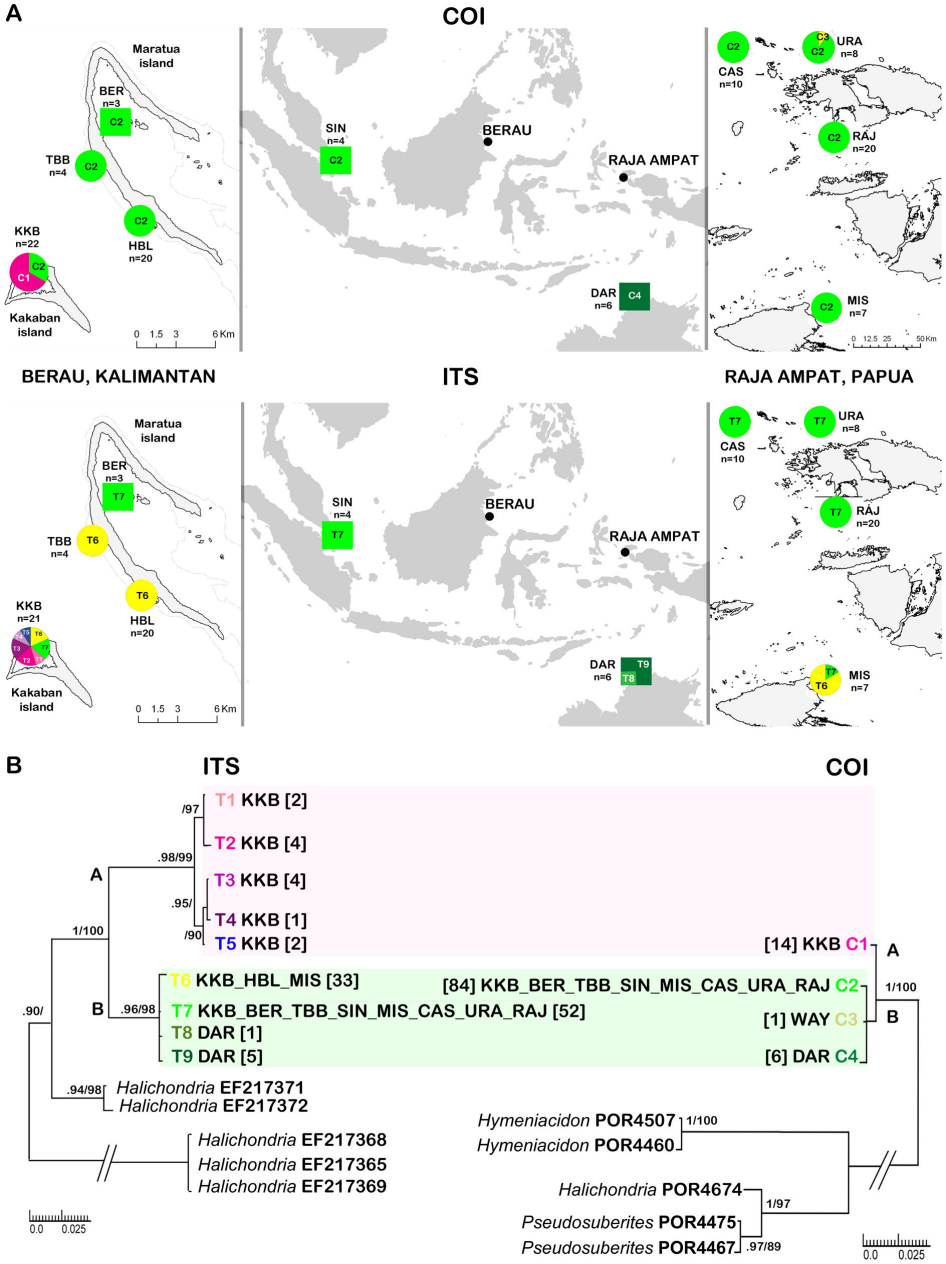
A Sample locations of the sponge *Suberites diversicolor*: top three maps represent distribution and frequencies of haplotypes for partial Cytochrome Oxidase I (COI) and bottom three maps of genotypes of internal transcribed spacer region (ITS) in Indonesia Singapore and Australia with insets of Berau (East Kalimantan left) and Raja Ampat (West Papua right) in Indonesia; location codes are explained in Table 2; circles represent marine lakes and squares are coastal populations; haplo/genotypes are indicated by number code (COI: C1-4 and ITS: T1-9) and color codes as provided in B. Note that scale differs per map. B Bayesian/maximum likelihood phylogram of 105 COI sequences (right) and 104 ITS sequences (left); each haplo/genotype indicated by specific color followed by location code and total number of samples in squared brackets. Only posterior probabilities of >90 and maximum likelihood values of >70 are indicated. Color blocks represent the same individuals for both molecular markers (i.e. lineage A (pink) and B (green) represented by the same individuals with both COI and ITS markers). Species of the family Halichondriidae were used for the outgroup followed by Genbank accession numbers. Scale bars indicate substitutions/site.

Here we have conducted the first phylogeographic study of Indonesian marine lake populations. The sponge species *Suberites diversicolor* [Porifera: Demospongiae: Suberitidae] is an ideal taxon to pursue this study as it allows comparison of multiple lakes at various scales and with varying degrees of connection to the sea (Fig. 1b). There are few other species that are prevalent in marine lakes [16]. *Suberites diversicolor* occurs in most moderately to highly isolated marine lakes in Indonesia [16], as well as in limited numbers of small populations in sheltered bays in Singapore, Indonesia and Australia [35]. This species shows great plasticity in adapting to harsh environments (low salinity and exposure to air) yet is absent in coral reefs [16], [19], [36], [37]. Sponges are one of the most dominant taxa in marine lakes in terms of biomass and species diversity [16], [17]. Recent comprehensive studies of sponge assemblages of marine lakes, coastal mangroves and coral reefs in Berau (East Kalimantan, Indonesia; Fig. 2) indicated that these lakes harbor a significantly different assemblage consisting of a subset of the fauna of the adjacent sea [21], [36]. Particularly the lake Kakaban harbors almost 33% of species not present in the surrounding [21]. The specific aims of this study were: 1) to estimate levels of diversity and divergence of seven marine lake populations and three coastal populations using two mitochondrial and two nuclear markers, 2) to study the phylogeography of *S. diversicolor* populations in marine lakes across Indonesia, 3) to investigate possible relationships between genetic diversity and the level of isolation of the lakes.

## Materials and Methods

### Permits

Indonesia: to L. Becking East Kalimantan IN 2008&2009: 0094/frp/sm/v/2009 and 1810/FRP/SM/VIII/2008; West Papua in 2011: 098/SIP/FRP/SM/V/2011.

Singapore: Singapore National Biodiversity Center collection permit to S.C. Lim,

Australia: Museum and Art Gallery of the Northern Territory permit to B. Alvarez.

### Sampling

Twenty four marine lakes and adjacent coastal habitats in Indonesia were thoroughly surveyed by snorkeling for the presence of the sponge *Suberites diversicolor*. Populations of *Suberites diversicolor* were located in seven marine lakes (29% of all surveyed lakes) in the region of Berau, East Kalimantan province (Kakaban lake, Haji Buang lake, Tanah Bamban lake) and the regions of Northern Raja Ampat (Cassiopeia lake, Urani lake, Sauwandarek lake) and Southern Raja Ampat (Misool Jellyfish lake) in West Papua province, and in mangroves along the coast of the island of Maratua in the region of Berau, East Kalimantan province (Fig. 2). Additional coastal populations were sampled from Johor Straight in Singapore (collected by S.C. Lim) and the man-made open Lake Alexander in Darwin, Australia (collected by B. Alvarez), resulting in a total of seven marine lake populations and three coastal populations sampled for this study (Fig. 2). The lakes Kakaban, Tanah Bamban, Haji Buang and Misool house immense perennial populations of the jellyfish *Mastigias papua* such as those that have been extensively documented in five marine lakes in Palau. For a full description of the sampled marine lakes, see Becking et al. [16]. The number of marine lakes worldwide is estimated at approximately 200 with clusters of ten or more lakes occurring in areas with a karstic limestone landscape such as Croatia, Bermuda, Vietnam, Palau, and Indonesia [20]. The lakes are formed in natural inland depressions and are subjected to a tidal regime, which is typically delayed in phase (ranging from 20 minutes to 4 hours) and dampened in amplitude (tide ranging from 20 cm to 1.5 m) compared to the adjacent sea [16], [17]. The level of obstruction of water exchange, i.e. the degree of isolation, differs per lake as does the salinity and environmental regimes within the lakes [14], [16]. The relative degree of isolation of each marine lake is provided in Table 2.

**Table 2:** Sample locations of ten *Suberites diversicolor* populations from marine lakes and coastal locations in Berau (East Kalimantan) and Raja Ampat (West Papua) in Indonesia, Darwin in Australia, and Singapore.

Code	Location	Region	Latitude	Longitude	Connection	density *S.diversicolor* (ind. 50m^2^)	color morphs *S.diversicolor*	nCOI	nITS	nCOII	n28S	Size lake (1000 m^3^)
*Lake*												
KKB	Kakaban lake	Berau	N02° 08′ 23.5″	E118° 30′ 31.9″	most isolated	15-Jan	green red	22	21	21	5	4000
HBL	Haji Buang lake	Berau	N02° 12′ 30.4″	E118° 35′ 40.8″	isolated	15–50	green, red, blue, purple, yellow	20	20	20	2	140
TBB	Tanah Bamban lake	Berau	N02° 13′ 50.0″	E118° 34′ 50.7″	least isolated	0–2		4	4	4	2	120
							green, red					
RAJ	Sauwandarek lake	Raja Ampat	S0° 35′ 19.6″	E130° 35′ 48.8″	very isolated	0–10	purple, blue green	21	21	21	2	84
CAS	Cassiopeia lake	Raja Ampat	N0° 08′ 36.6″	E130° 04′ 39.8″	least isolated	0–10	green	10	10	10	2	13
URA	Urani lake	Raja Ampat	N0° 06′ 05.1″	E130° 15′ 05.5″	isolated	0–2	green	8	8	8	1	68
MIS	Misool Jellfish Lake	Raja Ampat	S01° 55′	E130° 20′	isolated	0–2	green	7	7	7	1	12
*Coastal*												
BER	Maratua mangrove	Berau	N02° 12′ 52.3″	E118° 35′ 34.1″	open	0–1	green,yellow	3	3	3	1	
DAR	Lake Alexander Darwin	Australia	S12° 25′	E130° 50′	open	0–1	green	6	6	6	2	
SIN	Johor Strait	Singapore	N 01° 26′02.34″	E104°02′54.31″	open	0–1	purple, blue, green	4	4	4	2	

Per locality relative connection to the adjacent sea is provided and for the marine lake size. In addition the density of the target sponge species *Suberites diversicolor* color morphs and number of samples per genetic marker (COI, COII, 28S, ITS) is provided per location.

Collections were made randomly along the entire coastline of each of the lakes and specimens were collected at least 25 m distance from each other to avoid collecting clone siblings. Our aim was to collect 20 individuals per location, but in most locations the resident population size was too small to attain this target (see Table 2). Hence, sample sizes are small for some locations. The color and substrate of each specimen was recorded, and a photograph was taken either *in situ* or within 2 hours after collection. After collection, portions of the choanosome were cut into approximately 125 mm^3^ cubes, avoiding the surface to minimize potential contamination with protists or other sponge associates, and preserved in 96% ethanol, which was refreshed after 24 hours. The remainder of the samples were preserved in 70% ethanol and deposited in the Porifera collection of the Naturalis Biodiversity Center, The Netherlands (RMNH POR.) as voucher specimens. The investigated specimens are listed in the Appendix A.

### DNA Extraction, Amplification and Sequencing

Total DNA was extracted from 105 specimens using DNeasy tissue kit (Qiagen), following the instructions of the manufacturer. Partitions of four markers were amplified: two mitochondrial genes, cytochrome oxidase subunit 1 (COI) and subunit 2 (COII), and two nuclear markers, the nuclear ribosomal operons consisting of partial 18S rDNA, full-length internal transcribed spacer 1 and 2, 5.8S, and partial 28S rDNA fragments (ITS) and the D3–D5 region of the nuclear ribosomal 28S gene (28S). The nuclear markers are independent from the mitochondrial markers and therefore provide extra support in case of congruent results.

The standard DNA-barcoding fragment of COI was amplified by using a specific forward primer designed for *Suberites* SUB-COI-F: GGAATGATCGGGACAGCTTTTAGCATG and a degenerated reverse primer from Folmer et al. [38] designed by Meyer et al. [39]: dgHCO2198: TAA ACT TCA GGG TGA CCA AAR AAY CA. COII was amplified with the primers from Rua et al. [40]: CO2F: TTTTTCACGATCAGATTATGTTTA and CO2R: ATACTCGCACTGAGTTTGAATAGG. ITS amplified with primers from Wörheide (1998) RA2: GTCCCTGCCCTTTGTACACA and ITS2.2: CCTGGTTAGTTTCTTTTCCTCCGC. 28S was amplified in a subset of samples with primers from McCormack and Kelly (2002) RD3A: GACCCGTCTTGAAACACGA and RD5B2: ACACACTCCTTAGCGGA. Amplifications were carried out in 25 µl reaction volumes containing 5 µl Phire® Reaction Buffer,3 µl dNTPs (1 mM), 0.625 µl of each primer (10 µM), 0.25 µl Phire® Hotstart-*Taq* polymerase DNA (Thermo Scientific, Finnzymes), and 1 µl of DNA (10–20 ng/µl). The temperature regime for amplification: 94°C for 30 s; followed by 35 cycles of 94°C for 5 s; 50°C for 5 s; 72°C for 12 s; followed by 72°C for 1 min. PCR products were purified and sequenced by Macrogen Inc (Korea and The Netherlands).

### Data Analysis

The poriferan origin of the obtained sequences was verified through BLAST searches against Genbank (http://blast.ncbi.nlm.nih.gov/Blast.cgi). Sequences were handled in SEQUENCHER 4.10.1 (Gene Codes Corporation) and aligned with CLUSTALW and MUSCLE as implemented in DAMBE [41] and SEAview v 4.3.0 [42]. Alignment was conducted under default settings and optimized by eye. Alignments were collapsed to contain only unique sequence types in DAMBE. Haplo-/genotypes and nucleotide diversity as well as Tajima's D neutrality test were calculated per population with Arlequin v. 3.11 [43].

Phylogeographic analyses were carried out for COI and ITS. We used ITS outgroup sequences obtained from Genbank from the family Halichondriidae (Figure 1), as the available sequences for ITS of other Suberitidae were more distant than those from Halichondriidae. Several studies have shown that the families Suberitidae and Halichondriidae are sister groups [44], [45], [46]. To be consistent we also used species of the family Halichondriidae for the outgroup of the COI phylogram. The relatively best-fit DNA substitution model was selected by the Akaike Information Criterion deployed in jMODELTEST v. 0.1.1 [47] and this model (COI: HKY and ITS: GTR+G+I) was used for subsequent Bayesian and maximum likelihood phylogeny inferences. Phylogenetic reconstructions were performed under Bayesian inference criteria implemented in MrBayes v. 3.1.2. [48]. Each analysis consisted of two independent runs of four Metropolis-coupled Markov-chains, sampled at every 1,000^th^ generation at the default temperature (0.2). Analyses were terminated after the chains converged significantly as indicated by an average standard deviation of split frequencies <0.001. Convergence was also checked in Tracer v. 1.5.0 [49]. For comparison, maximum likelihood bootstrap analyses were conducted using MEGA v. 5.01 [50] using a heuristic search with 1,000 bootstrap replicates. The Bayesian and maximum likelihood phylograms were combined and visualized using TreeGraph 2 [51]. Within group *p*-distance (uncorrected), as well as net nucleotide divergence between groups were calculated in MEGA. A Kruskall-Wallis test was performed to test whether color or substrate preference significantly differed between lineages. To test for spatial structuring of samples we performed an analysis of molecular variance (AMOVA) and calculated pairwise *Φst* values between separate populations using Arlequin 3.5.1.2 [43]. Significance of pairwise *Φst* values (based on *p*-distances) was determined by 10,000 permutations and exact tests of population differentiation in Arlequin.

## Results

### Sequence variation (COI, COII, ITS, 28S)

All sequences were submitted to GenBank with accession numbers KF568951-KF568965 (Table S1). We obtained final alignments (excluding primers) for the sponge *S. diversicolor* of 519 bp for COI with four haplotypes (C1-4, 105 individuals, KF568960 - KF568963), 331 bp for COII with one haplotype (105 individuals, KF568964), 689 bp of ITS with nine genetic variants (T1-9, 104 individuals, KF568951- KF568959) (Table 2, Table S1). For a subset of 20 specimens we obtained 574 bp for 28S resulting in one genetic variant (KF568965).

### Divergent lineages in *S. diversicolor*

COI and ITS sequences were obtained from the same specimens and fall apart into two major lineages, termed A and B (Fig. 2B). These lineages represented reciprocally monophyletic groups for both markers and were strongly supported by both Bayesian and maximum likelihood inference methods (Fig. 2B). Lineage A was represented by haplotype C1 for COI and genotypes T1-5 for ITS. Lineage B is represented by COI haplotypes C2-4 and ITS genotypes T6-9 (Fig. 1B). Within lineage A there was no sequence variation in COI (n = 14), while the average *p*-distance within lineage B was 0.25% (n = 91). The net nucleotide divergence between lineage A & B for COI was 0.38%. Haplotype C1 (lineage A) differed by two basepairs from C2 (the dominant haplotype from lineage B) of which one resulted in a non-synonymous substitution between two unpolar amino acids, from isoleucine to valine. For ITS the average *p*-distance within lineage A was 0.44% (n = 13), while the average *p*-distance within lineage B was 0.29% (n = 91). The net nucleotide divergence between lineages A & B for ITS was 7.26%. Several indels of 1–3 bp length were observed and were consistent within lineages and differed between lineages. There were insertions in lineage A with respect to lineage B from 102–103 bp (either CT or TT), 380–381 bp (CA), 470–473 bp (GGA or GAA). There were gaps in lineage A with respect to lineage B from 139, 178–180, 549–555 bp. No double peaks were observed, and it we therefore assumed that no intragenomic polymorphisms occur within this species. The level of intragenomic polymorphisms differs per species [52]. We consider the risk of analyzing paralogous rDNA sequence types to be minimal as we see genealogical concordance across two unlinked loci. We did not detect a significant difference between lineage A & B in color (p = 0.249) or substrate preference (p = 0.100) using the independent samples Kruskal-Wallis Test.

### Diversity and spatial population structuring (COI & ITS)

Lineage A was only present in Kakaban lake while lineage B was present in all populations. The geographical distribution of COI haplotypes is shown in Fig. 2A. Of the four detected haplotypes in COI, haplotype C1 was restricted to Kakaban lake (East Kalimantan). Haplotype C3 only occurred in one individual in Urani lake (West Papua). The Darwin population was represented by haplotype C4, which was shared with no other population. Haplotype C2 was the most abundant haplotype, occurring in all populations except Darwin and was the dominant haplotype in the populations of Berau mangroves, Singapore, Sauwandarek lake, Cassopeia lake, Urani Lake, and Misool Jellyfish lake. Of the nine detected genotypes of ITS, five were restricted to Kakaban lake (genotypes T1-5), which are all representatives of lineage A. Genotype T7 (lineage B) was the most abundant and was shared by all sampled populations except Haji Buang Lake and Tanah Bamban lake (Kalimantan) and Darwin (Australia). Darwin was represented by the private genotypes T8-9. Haji Buang lake and Tanah Bamban lake harbored a single genotype (T6) that was shared by Kakaban lake (Kalimantan) and Misool Jellyfish lake (Papua).

Within lineage A in Kakaban lake there was only a single haplotype of COI while the ITS gene diversity was 0.8242+/- 0.0567, and ITS nucleotide diversity was 0.005656+/- 0.003392. Within lineage B all populations contained a single COI haplotype except Urani lake, which had two haplotypes with a haplotype diversity of 0.3333+/− 0.2152 and nucleotide diversity of 0.000624+/− 0.000822. For ITS, the majority of the populations contained only a single genotype, except for Kakaban lake, Darwin and Misool Jellyfish lake. The population in Kakaban lake had the highest gene and nucleotide diversity in lineage B, followed by Darwin and Misool Jellyfish lake (Table 3). Tajima's D tests of neutrality were carried out per population. The majority of the populations had a zero value due to the presence of only one genetic variant. Values of Tajima's D for ITS were negative, but not significant (p>0.1) in Misool lake and Darwin (Table 3).

**Table 3:** Genetic diversity indices based on ITS sequences per population of *Suberites diversicolor* of lineage A and B (location codes indicated in Table 2); gene diversity (*h*) nucleotide diversity (π) Tajima's D neutrality test.

Code	Lineage	n ITS	*h* ITS	π ITS	Tajima's D
KKB	A	13	0.8242+/−0.0567	0.005656+/−0.003392	1.3927
KKB	B	8	0.5357+/−0.1232	0.001578+/−0.001318	1.4488
HBL	B	20	0	0	0
TBB	B	4	0	0	0
RAJ	B	21	0	0	0
CAS	B	10	0	0	0
URA	B	8	0	0	0
MIS	B	7	0.2857+/−0.1964	0.000842+/−0.000879	−1.23716
BER	B	3	0	0	0
SIN	B	4	0	0	0
DAR	B	6	0.3333+/−0.2152	0.000980+/−0.000997	−1.13197

The majority of populations had only one haplotype resulting in 0 values for all indices calculated. All Tajima D values are not significant.

Spatial analysis of genetic structure of lineage B COI and ITS sequences showed that the Darwin population was strongly (*Φst* between 0.53–1) and significantly differentiated from all marine lakes populations (Table 4 & Table S2). Besides Darwin there was no significant differentiation in COI between the different populations (Table S2). The ITS marker was more diverse and showed more structure among the populations than COI (Fig. 1, Table 3). The Berau lakes (East Kalimantan), Kakaban and Haji Buang lakes were all significantly differentiated. The Raja Ampat lakes (West Papua) were not genetically differentiated from each other except Misool, which was differentiated from all Raja Ampat lakes, yet not from the populations of the lakes Kakaban, Haji Buang and Tanah Bamban (East Kalimantan). The AMOVA analyses revealed that significant portions of the total variance within lineage B can be attributed to differences among the following three groups 1. Berau coast, Singapore coast, Northern Raja Ampat lakes (Sauwandarek, Cassopeia, Urani), 2. Berau lakes (Kakaban, Tanah Bamban, Haji Buang), Southern Raja Ampat (Misool), 3. Darwin. The among group variation was 84.6% (p<0.001) and the within population variation was 10% (p<0.001).

**Table 4:** Pairwise *Φst* values between all populations of lineage B based on ITS sequences of *Suberites diversicolor* (location codes indicated in Table 2).

	KKB	HBL	TBB	RAJ	CAS	URA	MIS	BER	SIN
HBL	0.60591*								
TBB	0.30435	0							
RAJ	0.60591*	1*	1*						
CAS	0.4702*	1*	1*	0					
URA	0.42857	1*	1*	0	0				
MIS	0.13514	0.16749	−0.09804	0.91393*	0.86315*	0.84466*			
BER	0.25	1*	1*	0	0	0	0.76136*		
SIN	0.30435	1*	1*	0	0	0	0.78544*	0	
DAR	0.53451*	0.9512*	0.86348*	0.83584*	0.74359*	0.71049*	0.77327*	0.55882	0.60396

Values in bold and with asterisk indicate significant values (p<0.05).

## Discussion

### Divergent lineages in *S. diversicolor*

Two major lineages were uncovered in the populations of the sponge *S. diversicolor*. The congruent patterns of COI and ITS genetic markers and the degree of divergence between the two lineages (COI: 0.4% and ITS: 7.3%) are indicative of reproductive isolation, and thus we suggest that the two lineages (A and B) constitute different species. We searched for morphological and ecological characters to distinguish the two lineages, but did not find any. Genetic divergence can preclude morphological or ecological distinction. The skeletal structure and spicule lengths do not differ between lineages and fall within the natural variation of this species (see also [35]). The color and substrate preference are variable, but not consistent within a particular lineage. A related *Suberites* from Satonda lake (Sumbawa, Indonesia) displays different colors at different depths as a result of a symbiosis with the unicellular green algae *Chlorella* and symbiotic bacteria [53]. Phylogeographic studies in the Indo-Australian-Archipelago have uncovered numerous lineages in marine taxa that may represent undescribed cryptic species (e.g. [3], [6], [54]). Within sponges, molecular studies have revealed a high prevalence of morphologically cryptic sponge species (see review in [8], [55]).

The divergence between lineage A and B points to a long isolation in spite of the fact that they are sympatric in Kakaban lake (East Kalimantan). Within sponges there are several reports of sympatric cryptic species: *Tedania* spp. in mangroves [56], *Scopalina lophyropoda* [57], *Cliona* spp. [8], and *Hexadella* spp. [7]. Differential reproductive traits and output can promote the co-existence of sibling species (e.g. [57], [58]). This observation of divergent lineages in one lake is, however, not common in the phylogeographic studies conducted thus far on populations in the marine lakes in Palau [11], [22], [23], [59]. The Palauan studies on three distinct taxa (jellyfish, fish and bivalves) mostly show a pattern of one lineage occupying one lake [11], [22], [23], [59]. One reason why Kakaban lake may contain two lineages is the sheer size of the lake – at almost 4 km^2^ it is tenfold larger than any of the other marine lakes in Indonesia and the majority of Palau (Table 2; [15], [16]). Alternatively, lineage B could be a recent introduction to the lake. Sponge fragments are known to be transported by waterfowl [60] and workers from the neighboring island Maratua who stay on Kakaban for short periods to attend small crops may also act as possible vectors of *Suberites diversicolor* from the Maratua lakes or the mangroves near their village.

### Phylogeography

Lineage A is only present in Kakaban lake, while lineage B is present in all populations. Within lineage B the spatial genetic structure shows three groups: 1. the three Berau lakes and southern Raja Ampat lake, 2. Berau coast, Singapore coast and the three northern Raja Ampat lakes, 3. Darwin, Australia. At present there is no comprehensive phylogeographic study of sponges spanning the Indonesian archipelago, yet pronounced genetic differences in populations of other marine invertebrates and vertebrates are present between the Java Sea, the Indonesian Through Flow, and the seas of East Sulawesi [3], [5], [9], [61], [62]. The marine phylogeographic patterns of these studies strongly support the existence of a barrier in the area between the Sunda and Sahul shelves, where populations from Kalimantan are genetically isolated from those in Papua. Our data do not show such a clear East to West phylogeographic break. The Darwin population of the present study, though small in sample size, is genetically differentiated from the other populations. This is consistent with phylogeographic studies of sponges and other invertebrates that show a barrier within the Torres Strait (e.g. [63], [64]). Dispersal potential and habitat specialization may determine how lineages are distributed and how fauna of different geographic regions are connected (e.g. [9]). Many sponge population genetic and phylogeographic studies have revealed structured populations with in some cases evidence of (occasional) long distance dispersal events [65], [66], [67], [68], [69], [70]. This pattern is congruent with philopatric, shortlived larvae that recruit at short distances from the parental locations [71], [72], whilst at the same time the possibility of sponges to disperse as viable fragments in the currents or rafting on various floating material [73], [74], [75]. The reproductive cycle and larvae of *S. diversicolor* are unknown, but this species does produce asexual buds [Becking pers. obs.] which may survive a considerable amount of time in the plankton or by rafting before colonizing distant locations as proposed by Wörheide et al. [67] for *Leucetta chagosensis*.

For lineage B we found no private haplotypes in any of the Indonesian marine lakes, and many lakes were identical in composition. The only other phylogeographic studies on marine lakes have been in the islands of Palau on the jellyfish *Mastigias papua* [11]; [59], the fish *Sphaeramia orbicularis* [22], and the mussel *Brachidontes* sp. [23] (see Table 1). These studies show extreme genetic isolation, low genetic diversity, and in the cases of *Mastigias papua* and *Brachidontes* sp. rapid morphological evolution in the marine lakes [11], [22], [23], [59]. The lack of strong population structure between many of the Indonesian lakes of the present study may be caused by recurrent (recent and historic) gene flow among lakes. Alternatively, it is still possible that all these lakes are completely isolated, i.e. do not exchange any migrants, but that the lack of structure may be a result of the markers we used. These markers may not evolve fast enough for mutations to have accumulated to show the recent divergence. Of the four molecular markers used in the present study, ITS evolves the fastest and provided the highest resolution of spatial genetic structure. The difference in genetic diversity between COI and ITS is large. In contrast to most animals the mitochondrial DNA of sponges evolves slowly and generally slower than nuclear DNA [76], [77]. The interspecific variation of COI in sponges can be as low as 0–0.5% (*p*-distances) (e.g. [55], [78], [79]). However, in some sponge taxa COI can provide low but sufficient genetic variation within species over relatively short geographic distances [63], [69], [80].

We found no sequence variation in 28S and COII markers between any of the populations or between the two lineages of *S. diversicolor*. The D3–D5 region of the 28S fragment has been used to distinguish genera and species of a wide range of demosponge taxa including halichondrids [81], [82], but was also reported too conserved to discriminate between closely related species in other sponge taxa [7]. COII was proposed as a polymorphic mitochondrial marker for sponge phylogeography by Rua et al. [40]. Rua et al. [40] indicated that the variation of this marker could be low in halichondrid species *Hymeniacidon heliophila* but attributed their results to the collection of clone-mates. In the present study COII showed no variation between any of the samples spanning a wide geographic range. We conclude that COII is not a suitable marker for intraspecific variation or distinction between closely related species of the genus *Suberites* in particular, and probably more generally for the families Suberitidae and Halichondriidae.

### Isolation & genetic diversity

Kakaban lake is the largest and most isolated lake in Indonesia (see Table 2), and it houses a high proportion of endemic sponge species [16], [21]. In concordance, our study shows that the population of *Suberites diversicolor* displayed the highest genetic diversity with unique genetic variants that were not shared with two marine lakes at just 6 km distance (Fig. 1). These results indicate that Kakaban lake is very isolated both in physical and biological terms. Isolation acts to decrease the rate of immigration and thus to decrease the genetic diversity and the number of species expected at equilibrium in an island system [25], [26], [27], [83]. Yet isolation can also enhance species formation, with the diminished gene flow allowing populations to diverge and ultimately form new species if they remain isolated [17], [27], [83]. In Palau the degree of genetic distance between marine lake and adjacent sea populations was strongly correlated with the degree of connection from the lake to the sea and not the actual geographic distance between the populations [11], [23]. In the present study, the molecular markers used were not variable enough to detect a relationship between moderate levels of isolation and the genetic diversity of the lakes. For example, the populations in northern Raja Ampat lakes (West Papua) are not genetically differentiated, despite the limited physical connections to each other and to the adjacent sea.

### Biogeographic scenario

Kakaban lake was probably filled with sea water less than 12,000 years ago [13], [24]. Considering the deep divergence between lineages A & B in this lake, this divergence likely occurred well before the formation of Kakaban. Wörheide et al. [52] estimated an evolutionary rate of 1% per million years for ITS in a suberitid sponge *Prosuberites ‘laughlini’* based on the formation of Isthmus of Panama. Implementing the 1% mutational rate would mean that the two lineages diverged approximately 7 million years ago. Though this is a rough estimation with great error bars and rates of evolution may be higher for recently diverged lineages [84], the age is consistent with recent phylogeographic studies that suggest that many endemics from the Indo-Australian-Archipelago have origins in the early Pliocene-Miocene (3–20 million years ago; e.g. [85], [86], [87]).

Kakaban lake houses a genetic and species diversity of sponges, that appears to be absent from the surrounding sea (see also [21], [36]). Each lake is ephemeral, but the marine lakes ecosystem probably has occurred in various locations during the past glacial-cycles [24]. The Sunda shelf, which includes Borneo (Kalimantan), was exposed during the Last Glacial Maximum (LGM) when sea levels are estimated to have been approximately 110–140 m lower than modern sea levels [88], [89], [90]. Multiple larger and smaller depressions in the shelf have been recorded which presumably represented palaeo-lakes during the LGM [24] that could have become brackish marine lakes with the increase in sea level. During the LGM the Sunda Land region was also dominated by mangroves [91], and the water around the Sunda area would have been brackish due to the multiple river outlets [90]. These are both environments amenable for *S. diversicolor*. Ancient lineages/endemics may have ‘hopped’ from lake to lake or from mangrove to lake, as the lakes formed and subsequently disappeared with the rise and fall in sea level during the Plio-Pleistocene glacial cycles. Genetic signatures of glacial refugia are expected to be characterized by high genetic diversity and a mixture of ancestral and private haplotypes [92], [93]. While Kakaban matches this pattern, the lake could not have been a refugium during LGM (it was dry), however there may have been palaeo-lakes in the vicinity that served as such. Kakaban may be an area where multiple putative refugia populations have come into secondary contact, resulting in the high genetic diversity and the high number of endemics. Molecular studies on co-distributed taxa at larger scales including lakes from adjacent regions in Palau and Vietnam will enhance our understanding of the processes behind the unique marine lake diversity.

## Supporting Information

Table S1 List of *Suberites diversicolor* specimens studied. For each specimen the following information is provided: lineage to which it belongs (see Fig. 2) haplotype of Cytochrome Oxidase I (COI) GenBank Accession Number for the COI haplotype genotype of internal transcribed spacer region of nuclear ribosomal operons (ITS) GenBank Accession Number for ITS genotype location of collection (location codes indicated in Table 2) the color when alive substrate it resided on collection number within the Porifera Collection of the Naturalis Biodiversity Center (RMNH POR).
(XLSX)

Table S2 Pairwise *Φst* values between all populations of the sponge *Suberites diversicolor* lineage B based COI of *Suberites diversicolor* (location codes indicated in Table 2). Values in bold and with asterisk indicate significant values (p<0.05).
(XLSX)

## References

[1] Palumbi SR (1994) Genetics divergence reproductive isolation and marine speciation. Annual Review of Ecology & Systematics 25: 547–72.

[2] Knowlton N (2000) Molecular genetic analyses of species boundaries in the sea. Hydrobiologia 420: 73–90.

[3] Barber PH, Palumbi SR, Erdmann MV, Moosa MK (2002) Sharp genetic breaks among populations of *Haptosquilla pulchella* (Stomatopoda) indicate limits to larval transport: Patterns causes and consequences. Molecular Ecology 11: 659–674. 11972755 10.1046/j.1365-294x.2002.01468.x

[4] Peijnenburg KTCA, Breeuwer JAJ, Pierrot-Bults AC, Menken SBJ (2004) Phylogeography of the planktonic chaetognath *Sagitta setosa* reveals isolation in European seas. Evolution 58: 1472–1487. 15341150 10.1111/j.0014-3820.2004.tb01728.x

[5] Nuryanto A, Kochzius M (2009) Highly restricted gene flow and deep evolutionary lineages in the giant clam *Tridacna maxima* . Coral Reefs 28: 607–619.

[6] Malay MCD, Paulay G (2010) Peripatric speciation drives diversification and distributional pattern of reef hermit crabs (Decapoda: Diogenidae: *Calcinus*). Evolution 64: 634–662. 19796150 10.1111/j.1558-5646.2009.00848.x

[7] Reveillaud J, Remerie T, van Soest R, Erpenbeck D, Cárdenas P, et al (2010) Species boundaries and phylogenetic relationships between Atlanto-Mediterranean shallow-water and deep-sea coral associated *Hexadella* species (Porifera Ianthellidae). Molecular Phylogenetics and Evolution 56: 104–114. 20382244 10.1016/j.ympev.2010.03.034

[8] Xavier JR, Rachello-Dolmen PG, Parra-Velandia F, Schoenberg CHL, Breeuwer JAJ, et al (2010) Molecular evidence of cryptic speciation in the “cosmopolitan” excavating sponge *Cliona celata* (Porifera Clionaidae). Molecular Phylogenetics and Evolution 56: 13–20. 20363344 10.1016/j.ympev.2010.03.030

[9] Carpenter KE, Barber PH, Crandall ED, Ablan-Lagman CA, Ambariyanto Mahardika GA, et al. (2011) Comparative phylogeography of the Coral Triangle and implications for marine management. Journal of Marine Biology: Article ID 396982, doi:10.1155/2011/396982

[10] Peijnenburg KTCA, Goetze E (2013) High evolutionary potential of marine zooplankton. Ecology and Evolution 3: 2765–2781. 24567838 10.1002/ece3.644 PMC3930040

[11] Dawson MN, Hamner WM (2005) Rapid evolutionary radiation of marine zooplankton in peripheral environments. Proceedings of the National Academy of Sciences 102: 9235–9240. 10.1073/pnas.0503635102 PMC1166623 15964980

[12] Holthuis LB (1973) Caridean shrimps found in land-locked saltwater pools at four Indo-West Pacific localities (Sinai Peninsula Funafuti atoll Maui and Hawaii Islands) with the description of one new genus and four new species. Zoologische Verhandelingen Leiden 128: 1–48.

[13] Dawson MN (2006) Island evolution in marine lakes. JMBA Global Marine Environment: 26–29.

[14] Hamner WM, Hamner PP (1998) Stratified marine lakes of Palau (Western Caroline islands). Physical Geography 19: 175–220.

[15] Colin PL (2009) Marine environments of Palau. Indo-Pacific Press San Diego, 416.

[16] Becking LE, Renema W, Santodomingo NK, Hoeksema BW, Tuti Y, et al (2011) Recently discovered landlocked basins in Indonesia reveal high habitat diversity in anchialine systems. Hydrobiologia 677: 89–105.

[17] Tomascik T, Mah AJ (1994) The ecology of ‘*Halimeda* lagoon”: An anchialine lagoon of a raised atoll Kakaban island East Kalimantan Indonesia. Tropical Biodiversity 2: 385–399.

[18] Tomascik T, Mah AJ, Nontji A, Moosa MK (1997) The Ecology of the Indonesia Seas. Part II. Periplus Singapore, 752.

[19] Azzini F, Calcinai B, Cerrano C, Bavestrello G, Pansini M (2007) Sponges of the marine karst lakes and of the coast of the islands of Ha Long Bay (North Vietnam). In: Custodia MR, Lobo-Hajdu G, Hajdu E, Muricy G, Porifera research: Biodiversity innovation and sustainability. Rio de Janeiro, 157–164.

[20] Dawson MN, Martin LE, Bell LJ, Patris S (2009) Marine lakes. In: Gillespie R, ClagueDA, Encyclopedia of islands. University of California Press Berkeley, 603–607.

[21] Becking LE, Cleary DFR, de Voogd NJ (2013) Sponge species composition abundance and cover in marine lakes and coastal mangroves of Berau Indonesia. Marine Ecology Progress Series 481: 105–120.

[22] Gotoh RO, Sekimoto H, Chiba SN, Hanzawa N (2009) Peripatric differentiation among adjacent marine lake and lagoon populations of a coastal fish *Sphaeramia orbicularis* (Apogonidae Perciformes Teleostei). Genes & Genetic Systems 84: 287–295. 20057166 10.1266/ggs.84.287

[23] Goto TV, Tamate HB, Hanzawa N (2011) Phylogenetic characterization of three morphs of mussels (Bivalvia Mytilidae) inhabiting isolated marine environments in Palau islands. Zoological Science (Tokyo) 28: 568–579. 10.2108/zsj.28.568 21800997

[24] Sathiamurthy E, Voris HK (2006) Maps of Holocene sea level transgression and submerged lakes on the Sunda shelf. The Natural History Journal of Chulalongkorn University Supplement 2: 1–44.

[25] MacArthur RH, Wilson EO (1967) The theory of island biogeography. Princeton University Press, 203.

[26] Whittaker RJ, Fernandez-Palacios JM (2008) Island biogeography. Ecology Evolution and Conservation. 2^nd^ Edition Oxford University Press, 416.

[27] Rosindell J, Hubbell SP, Etienne RS (2011) The unified neutral theory of biodiversity and biogeography at age ten. Trends in Ecology & Evolution 26: 340–348. 21561679 10.1016/j.tree.2011.03.024

[28] Santos SR (2006) Patterns of genetic connectivity among anchialine habitats: A case study of the endemic Hawaiian shrimp *Halocaridina rubra* on the island of Hawaii. Molecular Ecology 15: 2699–2718. 16911195 10.1111/j.1365-294X.2006.02965.x

[29] Craft JD, Russ AD, Yamamoto MN, Iwai TY, Hau S, et al (2008) Islands under islands: The phylogeography and evolution of *Halocaridina rubra* Holthuis 1963 (Crustacea: Decapoda: Atyidae) in the Hawaiian archipelago. Limnology and Oceanography 53: 675–689.

[30] Page TJ, Humphreys WF, Hughes JM (2008) Shrimps down under: Evolutionary relationships of subterranean crustaceans from Western Australia (Decapoda: Atyidae: Stygiocaris). PLoS ONE 3: :e16181611–1612. 10.1371/journal.pone.0001618 PMC2229661 18286175

[31] Botello A Alvarez F (2010) Genetic variation in the stygobitic shrimp *Creaseria morleyi* (Decapoda: Palaemonidae) evidence of bottlenecks and re-invasions in the Yucatan peninsula. Biological Journal of the Linnean Society 99: 315–325.

[32] Bauzà-Ribot MM, Jaume D, Fornós JJ, Juan C, Pons J (2011) Islands beneath islands: Phylogeography of a groundwater amphipod crustacean in the Balearic archipelago. BMC Evolutionary Biology 11: 221. 21791038 10.1186/1471-2148-11-221 PMC3161010

[33] Russ A, Santos SR, Muir C (2010) Genetic population structure of an anchialine shrimp *Metabetaeus lohena* (Crustacea: Alpheidae) in the Hawaiian Islands. Revista de Biologia Tropical 58: 159–170. 20411714 10.15517/rbt.v58i1.5201

[34] Kano Y, Kase T (2004) Genetic exchange between anchialine cave populations by means of larval dispersal: The case of a new gastropod species *Neritilia cavernicola* . Zoologica Scripta 33: 423–437.

[35] Becking LE, Lim SC (2009) A new *Suberites* (Demospongiae: Hadromerida: Suberitidae) from the tropical Indo-West Pacific. Zoologische Mededelingen (Leiden) 83: 853–862.

[36] de Voogd NJ, Becking LE, Cleary DFR (2009) Sponge community composition in the Derawan islands ne Kalimantan Indonesia. Marine Ecology Progress Series 396: 169–180.

[37] Lim S-C, de Voogd NJ, Tan K-S (2009) Fouling sponges (Porifera) on navigation buoys from Singapore waters. Raffles Bulletin of Zoology: 41–58.

[38] Folmer O, Black M, Hoeh W, Lutz R, Vrijenhoek R (1994) DNA primers for amplification of mitochondrial cytochrome c oxidase subunit I from diverse metazoan invertebrates. Molecular Marine Biology and Biotechnology 3: 294–299. 7881515

[39] Meyer CP, Geller JB, Paulay G (2005) Fine scale endemism on coral reefs: Archipelagic differentiation in turbinid gastropods. Evolution 59: 113–125. 15792232

[40] Rua CPJ, Zilberberg C, Sole-Cava AM (2011) New polymorphic mitochondrial markers for sponge phylogeography. Journal of the Marine Biological Association UK 91: 1015–1022.

[41] Xia X, Xie Z (2001) Dambe: Software package for data analysis in molecular biology and evolution. Journal of Heredity 92: 371–373. 11535656 10.1093/jhered/92.4.371

[42] Gouy M, Guindon S, Gascuel O (2010) Seaview version 4: A multiplatform graphical user interface for sequence alignment and phylogenetic tree building. Molecular Biology and Evolution 27: 221–224. 19854763 10.1093/molbev/msp259

[43] Excoffier L, Lischer HEL (2010) Arlequin suite ver 3.5: A new series of programs to perform population genetics analyses under linux and windows. Molecular Ecology Resources 10: 564–567. 21565059 10.1111/j.1755-0998.2010.02847.x

[44] Chombard C, Boury-Esnault N, Tillier S (1998) Reassessment of homology of morphological characters in tetractinellid sponges based on molecular data. Systematic Biology 47: 351–366. 12066683 10.1080/106351598260761

[45] Chombard C, Boury-Esnault N (1999) Good congruence between morphology and molecular phylogeny of Hadromerida or how to bother sponge taxonomists. Memoirs of Queensland Museum 44: 100.

[46] Morrow CC, Picton BE, Erpenbeck D, Boury-Esnault N, Maggs CA, et al (2012) Congruence between nuclear and mitochondrial genes in Demospongiae: A new hypothesis for relationships within the G4 clade (Porifera: Demospongiae). Molecular Phylogenetics and Evolution 62: 174–190. 22001855 10.1016/j.ympev.2011.09.016

[47] Posada D 2008. Jmodeltest: Phylogenetic model averaging. Molecular Biology and Evolution 25: 1253–1256. 18397919 10.1093/molbev/msn083

[48] Huelsenbeck JP, Ronquist F (2001) Mrbayes: Bayesian inference of phylogenetic trees. Bioinformatics 17: 754–755. 11524383 10.1093/bioinformatics/17.8.754

[49] Rambaut A, Drummond AJ (2007) Tracer v1.4 available from http://beast.Bio.Ed.Ac.Uk/tracer.

[50] Tamura K, Peterson D, Peterson N, Stecher G, Nei M, et al (2011) Mega5: Molecular evolutionary genetics analysis using maximum likelihood evolutionary distance and maximum parsimony methods. Molecular Biology and Evolution 28: 2731–2739. 21546353 10.1093/molbev/msr121 PMC3203626

[51] Stöver BC, Müller KF (2010) TreeGraph 2: Combining and visualizing evidence from different phylogenetic analyses. BMC Bioinformatics 11: 7. 20051126 10.1186/1471-2105-11-7 PMC2806359

[52] Wörheide G, Nichols SA, Goldberg J (2004) Intragenomic variation of the rDNA internal transcribed spacers in sponges (phylum Porifera): Implications for phylogenetic studies. Molecular Phylogenetics and Evolution 33: 816–830. 15522806 10.1016/j.ympev.2004.07.005

[53] Arp G, Reitner J, Wörheide G, Landmann G (1996) New data on microbial communities and related sponge fauna from the alkaline Satonda Crater Lake (Sumbawa Indonesia). In: Reitner J Neuweiler F &GunkelF, : Global and Regional Controls on Biogenetic Sedimentation. I. Reef Evolution. Research Reports. - Göttinger Arb. Geol. Paläont Sonderband 2: : 1–7Göttingen.

[54] Crandall ED, Jones ME, Munoz MM, Akinronbi B, Erdmann MV, et al (2008) Comparative phylogeography of two seastars and their ectosymbionts within the Coral Triangle. Molecular Ecology 17: 5276–5290. 19067797 10.1111/j.1365-294X.2008.03995.x

[55] Uriz MJ, Turon X (2012) Sponge ecology in the molecular era. Advances in Marine Biology 61: 345–410. 22560781 10.1016/B978-0-12-387787-1.00006-4

[56] Wulff JL (2006) Sponge systematics by starfish: Predators distinguish cryptic sympatric species of Caribbean fire sponges *Tedania ignis* and *Tedania klausi* n. sp (Demospongiae Poecilosclerida). Biological Bulletin 211: 83–94. 16946245 10.2307/4134581

[57] Blanquer A, Uriz M-J, Agell G (2008) Hidden diversity in sympatric sponges: Adjusting life-history dynamics to share substrate. Marine Ecology Progress Series 371: 109–115.

[58] Pérez-Porro A-R, González J, Uriz MJ (2012) Reproductive traits explain contrasting ecological features in sponges: The sympatric poecilosclerids *Hemimycale columella* and *Crella elegans* as examples. Hydrobiologia 687: 315–33.

[59] Dawson MN (2005) Five new subspecies of *Mastigias* (Scyphozoa: Rhizostomeae:Mastigiidae) from marine lakes Palau Micronesia. Journal of the Marine Biological Association UK 85: 679–694.

[60] Pronzato R, Manconi R (1994) Adaptive strategies of sponges in inland waters. Bolletino di Zoologia 61: 395–401.

[61] Barber PH, Palumbi SR, Erdmann MV (2006) Comparative phylogeography of three co-distributed stomatopods: Origins and timing of regional lineage diversification in the coral triangle. Evolution 60: 1825–1839. 17089967

[62] Timm J, Kochzius M (2008) Geological history and oceanography of the Indo-Malay archipelago shape the genetic population structure in the false clown anemonefish (*Amphiprion ocellaris*). Molecular Ecology 17: 3999–4014. 19238702 10.1111/j.1365-294x.2008.03881.x

[63] Andreakis N, Luter HM, Webster N (2012) Cryptic speciation and phylogeographic relationships in the elephant ear sponge *Ianthella basta* (Porifera Ianthellidae) from northern Australia. Zoological Journal of the Linnean Society 166: 225–235.

[64] Vogler C, Benzie JAH, Tenggardjaja K, Barber PH, Wörheide G (2013) Phylogeography of the crown-of-thorns starfish: genetic structure within the Pacific species. Coral Reefs 32: 515–525.

[65] Wörheide G, Hooper JNA, Degnan BM (2002) Phylogeography of western Pacific *Leucetta ‘chagosensis’* (Porifera: Calcarea) from ribosomal DNA sequences: Implications for population history and conservation of the great barrier reef world heritage area (Australia). Molecular Ecology 11: 1753–1768. 12207725 10.1046/j.1365-294x.2002.01570.x

[66] Wörheide G, Sole-Cava AM, Hooper JNA (2005) Biodiversity molecular ecology and phylogeography of marine sponges: Patterns implications and outlooks. Integrative and Comparative Biology 45: 377–385. 21676783 10.1093/icb/45.2.377

[67] Wörheide G, Epp LS, Macis L (2008) Deep genetic divergences among indo-pacific populations of the coral reef sponge *Leucetta chagosensis* (Leucettidae): Founder effects vicariance or both? BMC Evolutionary Biology 8: 24. 18221552 10.1186/1471-2148-8-24 PMC2267160

[68] Lopez-Legentil S, Pawlik JR (2009) Genetic structure of the caribbean giant barrel sponge *Xestospongia muta* using the I3-m11 partition of COI. Coral Reefs 28: 157–165.

[69] DeBiasse MB, Richards VP, Shivji MS (2010) Genetic assessment of connectivity in the common reef sponge *Callyspongia vaginalis* (Demospongiae: Haplosclerida) reveals high population structure along the Florida reef tract. Coral Reefs 29: 47–55.

[70] Xavier JR, van Soest RWM, Breeuwer JA, Martins AMF, Menken SBJ (2010) Phylogeography, genetic diversity and structure of the Poecilosclerid sponge *Phorbas fictitius* at oceanic islands. Contributions to Zoology 79: 19–129.

[71] Mariani S, Uriz MJ, Turon X (2005) The dynamics of sponge larvae assemblages from northwestern Mediterranean nearshore bottoms. Journal of Plankton Research 27: 249–262.

[72] Mariani S, Uriz M-J, Turon X, Alcoverro T (2006) Dispersal strategies in sponge larvae: Integrating the life history of larvae and the hydrologic component. Oecologia 149: 174–184. 16710659 10.1007/s00442-006-0429-9

[73] Wulff JL (1991) Asexual fragmentation genotype success and population-dynamics of erect branching sponges. Journal of Experimental Marine Biology and Ecology 149: 227–247.

[74] Wulff JL (1995) Effects of a hurricane on survival and orientation of large erect coral-reef sponges. Coral Reefs 14: 55–61.

[75] Maldonado M Uriz M.J 1999. Sexual propagation by sponge fragments. Nature 398: 476–476.

[76] Shearer TL, Van Oppen MJ, Romano SL, Wörheide G (2002) Slow mitochondrial DNA sequence evolution in the Anthozoa (Cnidaria). Molecular Ecology 11: 2475–2487. 12453233 10.1046/j.1365-294x.2002.01652.x

[77] Hellberg ME (2006) No variation and low synonymous substitution rates in coral mtDNA despite high nuclear variation. BMC Evolutionary Biology 6: 24. 16542456 10.1186/1471-2148-6-24 PMC1431588

[78] Wörheide G (2006) Low variation in partial cytochrome oxidase subunit I (COI) mitochondrial sequences in the coralline demosponge Astrosclera willeyana across the Indo-Pacific. Marine Biology 148: 907–912.

[79] Pöppe J, Sutcliffe P, Hooper JNA, Wörheide G, Erpenbeck D (2011) COI barcoding reveals new clades and radiation patterns of Indo-Pacific sponges of the family Irciniidae (Demospongiae: Dictyoceratida). PLoS ONE 5(4): e9950 doi:10.1371/journal.pone.0009950 10.1371/journal.pone.0009950 PMC2848591 20376349

[80] Duran S, Rützler K (2006) Ecological speciation in a Caribbean marine sponge. Molecular Phylogenetics and Evolution 40: 292–297. 16574436 10.1016/j.ympev.2006.02.018

[81] McCormack GP, Kelly M (2002) New indications of the phylogenetic affinity of *Spongosorites suberitoides* Diaz et al 1993 (Porifera Demospongiae) as revealed by 28s ribosomal DNA. Journal Natural History 36: 1009–1021.

[82] Erpenbeck D, Breeuwer JAJ, van Soest RWM (2005) Implications from a 28s rRNA gene fragment for the phylogenetic relationships of halichondrid sponges (Porifera: Demospongiae). Journal Zoological Systematics and Evolutionary Research 43: 93–99.

[83] Chen X-Y, He F (2009) Speciation and endemism under the model of island biogeography. Ecology 90: 39–45. 19294911 10.1890/08-1520.1

[84] Ho SYW, Lanfear R, Bromham L, Phillips MJ, Soubrier J, et al (2011) Time-dependent rates of molecular evolution. Molecular Ecology 20: 3087–3101. 21740474 10.1111/j.1365-294X.2011.05178.x

[85] Renema W, Bellwood DR, Braga JC, Bromfield K, Hall R, et al (2008) Hopping hotspots: Global shifts in marine biodiversity. Science 321: 654–657. 18669854 10.1126/science.1155674

[86] Bellwood DR, Meyer CP (2009) Searching for heat in a marine biodiversity hotspot. Journal of Biogeography 36: 569–576.

[87] Cowman PF, Bellwood DR (2011) Coral reefs as drivers of cladogenesis: Expanding coral reefs cryptic extinction events and the development of biodiversity hotspots. Journal of Evolutionary Biology 24: 2543–2562. 21985176 10.1111/j.1420-9101.2011.02391.x

[88] Geyh MA, Kudrass HR, Streif H (1979) Sea level changes during the late Pleistocene and Holocene in the strait of Malacca. Nature 278: 441–443.

[89] Voris HK (2000) Maps of Pleistocene sea levels in Southeast Asia: Shorelines river systems and time durations. Journal of Biogeography 27: 1153–1167.

[90] Hoeksema B (2007) Delineation of the Indo-Malayan centre of maximum marine biodiversity: The coral triangle. In: Renema W, Biogeography in time and place: Distributions, barriers and islands. Vol 29 . Springer Netherlands, 117–178

[91] Morley RJ (2000) Origin and Evolution of Tropical Rain Forests. John Wiley & Sons Ltd, 378.

[92] Hewitt G (2000) The genetic legacy of the quaternary ice ages. Nature 405: 907–913. 10879524 10.1038/35016000

[93] Maggs CA, Castilho R, Foltz D, Henzler C, Jolly MT, et al (2008) Evaluating signatures of glacial refugia for north Atlantic benthic marine taxa. Ecology 89: S108–S122. 19097488 10.1890/08-0257.1

[94] Santos SR, Weese DA (2011) Rocks and Clocks: Linking geologic history and rates of genetic differentation in anchialine organisms. *Hydrobiologia* . 677: 54–63.

